# Crushing Characteristics Comparison between Aluminum/CFRP and Aluminum/CFRP/Aluminum Hybrid Tubes

**DOI:** 10.3390/polym14194034

**Published:** 2022-09-26

**Authors:** Fei Ge, Yuan Lin, Fukai Zhang, Zhongwei Zhang, Mingyang Wang

**Affiliations:** 1 State Key Laboratory for Disaster Prevention & Mitigation of Explosion & Impact, Army Engineering University of PLA, Nanjing 210000, China; 2 Field Engineering College, Army Engineering University of PLA, Nanjing 210000, China

**Keywords:** aluminum/CFRP hybrid tubes, axial crushing behavior, geometry size, experiment

## Abstract

This study experimentally investigated the axial crushing characteristics of the hybrid tubes with the configuration of aluminum/carbon fiber-reinforced polymer (CFRP) (1/1) and aluminum/CFRP/aluminum (2/1). The effects of geometry size and fiber lay-up sequence on the axial crushing energy-absorption performances and failure modes of the two types of hybrid tubes were compared. The results showed that the energy absorption of the specimens with [0°/90°] lay-up sequence was better than that of the ones with [45°/−45°] lay-up sequence for both types of hybrid tubes. The proper length of the tubes should be selected to avoid too small a length-to-diameter ratio so that a stable and controllable progressive crushing failure mode can be achieved. When the crushing failure process was relatively stable, the specific energy absorption and crushing force efficiency of the 2/1 hybrid tubes were not affected by the geometric size. The energy absorption of the hybrid tubes was higher than the sum of the energy absorption of all the corresponding individual tubes, showing a positive hybrid effect.

## 1. Introduction

Energy-absorbing components are commonly used in engineering structures [1,2]; for example, they are used in the front anti-collision devices of a bridge [3], in high-speed rail [4], aircraft bottom landing devices, or buffer structures under cabin floors [5]. Currently, metal or fiber-reinforced polymer (FRP) is the main material used for producing energy-absorbing components. However, it is difficult to satisfy modern engineering lightweight and high-strength requirements because of the high density and poor load-bearing capacity of traditional metal materials. FRP is highly sensitive to impact damage because of its elastic brittleness, which considerably lowers the residual load-bearing capacity of FRP after an impact [6,7,8]. Additionally, advanced high-performance FRP (e.g., carbon fiber-reinforced polymer, CFRP) is more expensive than traditional metal materials [9], making it difficult to replace traditional metal materials with FRP on a large scale. Therefore, it is both economical and efficient to combine FRP materials with traditional metal materials to form metal/FRP components.

Understanding how to make metal/CFRP components function together under load through a suitable manufacturing process is crucial for producing metal/CFRP components. The simplest method for placing FRP outside a metal layer is to paste FRP on the outer surface of the metal layer by hand lay-up. FRP reinforcing layers and metal components are typically bonded by epoxy resin adhesive. However, it is necessary to examine the production quality of metal/CFRP components because of the lack of curing pressure. Kim et al. [10,11] used an autoclave to prepare aluminum alloy/FRP hybrid square tubes. The authors used the hand lay-up method to paste the outer FRP layer and placed the layer in a silica gel mold. The silica gel mold expanded when the layer was heated and cured, giving the FRP layer sufficient curing pressure. Sun et al. [12] used wet filament winding to prepare aluminum/FRP hybrid circular tubes. The technique involved the use of mechanized methods to generate an outer FRP layer when the FRP was attached to the interior of the metal layer. Lee et al. [13] applied curing pressure using mechanical pressure to ensure that the FRP layer was attached to the inner wall of a metal tube. Zhu et al. [14] prepared similar hybrid circular tubes by combining the expansion of internal airbags with the pressure applied by an external press. Recent new processes have enabled the production of multilayer metal/FRP hybrid tubes. Yang et al. [15] rolled an aluminum alloy sheet and CFRP prepreg together on a bar to form a circular tube and then used a vacuum pressure as the curing pressure to produce multilayer metal/CFRP hybrid tubes. Han et al. [16] used the spinning process to prepare CFRP/aluminum hybrid tubes.

Energy-absorbing components mainly support transverse [17,18], inclined [19,20], and axial loads [21,22] and are typically used for supporting axial crushing loads. Recently, many studies have investigated the axial crushing energy absorption of 1/1 metal/FRP components because of the early development of 1/1 metal/FRP components and the relatively developed manufacturing process. Experimental studies have thoroughly examined the effects of fiber lay-up sequence and thickness [10] as well as interface properties [23,24] and stacking configuration [14] on the crushing performance of metal/FRP hybrid tubes and analyzed the failure mode and crushing energy-absorption parameters of metal/FRP hybrid tubes. Some thorough numerical simulation studies were also conducted, and the studies can be divided into two categories: the comparison of modeling strategies of crushing finite element model [25,26,27] and the optimization of parameters of the corresponding individual tubes using the finite element model [19,28,29]. Most studies have focused on the theoretical model prediction of the mean crushing force [30,31,32]. Recently, studies have investigated multilayer hybrid tubes and found the axial energy-absorption potential of the tube because of the gradual development of the multilayer hybrid metal/FRP tube process [16,33,34,35]. However, researchers have not investigated the influence of basic parameters such as geometric size, lay-up sequence on the crushing energy-absorption performance, and failure mode of multilayer hybrid tube with high manufacturing quality; the relative lack of investigations is because research on multilayer hybrid tubes is still in the preliminary development stage.

We experimentally investigated 1/1 and 2/1 hybrid tubes, compared the deformation and energy-absorption characteristics of the two hybrid tube types and their corresponding individual tubes, and identified the energy dissipation mechanism of both hybrid tube types and their corresponding individual tubes. Additionally, we examined the influence of geometric size, lay-up sequence, and other parameters on the energy-absorption performance and crushing failure mode of both hybrid tube types.

## 2. Material and Experimental Procedure

### 2.1. Material Fabrication

**Figure 1:**
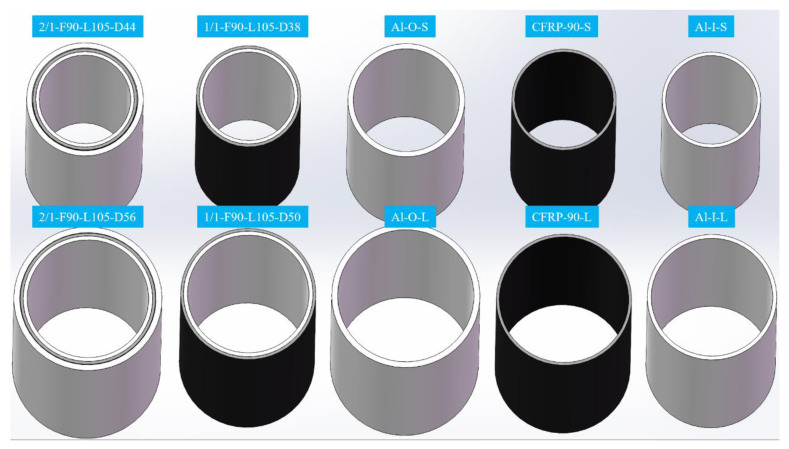
Two types of aluminum/CFRP hybrid tubes and three kinds of corresponding individual tubes.

**Figure 2:**
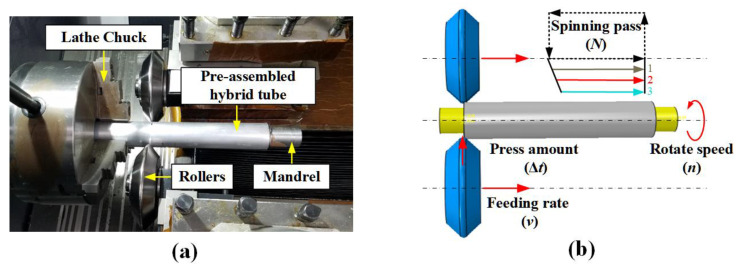
Power spinning process: (**a**) experimental set-up; (**b**) diagram of forming parameters.

**Figure 3:**
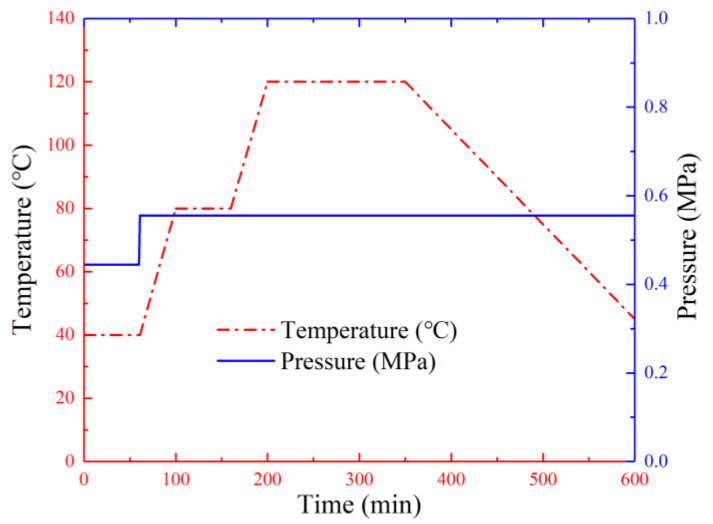
Curing procedure for the hybrid tubes.

T300/7901 unidirectional CFRP prepregs (thickness of 0.15 mm, 60% fiber volume fraction), 6517A epoxy resin film (thickness of 0.15 mm) provided by Weihai Guangwei Group (Wehai, Shandong, China) and 6061 seamless aluminum tubes provided by Guangdong Kepin Group (Shenzhen, Guangdong, China) were applied as raw materials. Figure 1 illustrates two types of aluminum/CFRP hybrid tubes with 1/1 and 2/1 layout configurations and corresponding aluminum and CFRP tubes. The 1/1 hybrid tube was fabricated by sequentially wrapping epoxy film and CFRP layers onto the aluminum tube according to the designed lay-up configuration. The 2/1 hybrid tube was prepared by power spinning process, which is schematically shown in Figure 2. Phosphoric acid anodizing method according to HB/Z 1987–1991 standard was first employed on the aluminum tubes to promote bonding effects between aluminum and CFRP layers. Then, the component layers were pre-assembled according to the lay-up configuration and mounted on the power spinning machine. During fabrication, the pre-assembled hybrid tube and mandrel rotated at 500 r/min, which drove the two rollers by friction to rotate at their own axis. The rotary rollers gradually extruded the outer aluminum tube along the axial direction. Through previous trials, the axial feeding rate was set as 200 mm/min and radial press amount was chosen as 0.7 mm. Three spinning passes were demanded to achieve the target press amount, as the thickness of the outer aluminum tube could be reduced at a small degree per pass to improve the forming stability and quality. Finally, both 1/1 and 2/1 hybrid tubes were cured in a vacuum oven based on the curing procedure in Figure 3. Table 1 presents the geometric parameters of the two hybrid tubes and their corresponding individual tubes (6061 aluminum alloy). The hybrid tubes are named in the order of “stacking configuration-F angle of fiber layer-L length of specimen-D outer diameter of specimen”. The four aluminum alloy tubes and two CFRP tubes listed in the table are the corresponding individual tubes of the two 2/1 hybrid tubes with different outer diameters, respectively.

### 2.2. Experimental Procedures

**Figure 4:**
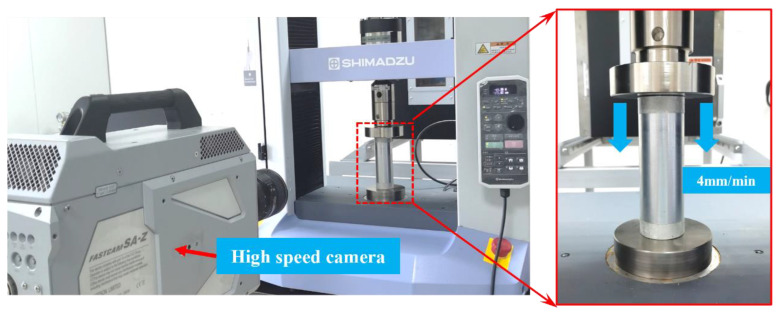
Quasi-static axial crushing test set-up.

The axial crushing tests were conducted on a Shimadzu universal testing machine, as shown in Figure 4. The specimen was axially compressed at a constant platen speed of 4 mm/min, during which process the compressive load and axial displacement were recorded by the testing machine. In order to capture the crushing failure process of the specimens, a Fastcam SA-Z was employed. The final crushing distance was two-thirds of the specimen’s original length to fully exploit the crushing characteristics of the hybrid tubes.

### 2.3. Crashworthiness Indicators

Typical indexes used for evaluating the energy-absorption performance mainly include the following:

**Total energy absorption (*EA*):** This is the total energy absorbed during the crushing test. The total energy absorption is used for determining the energy-absorption capacity and is expressed as:

(1) $$EA=\int_{0}^{d}F(x)dx$$

where *d* is the total crushing displacement, and *F*(*x*) is the instantaneous crushing force at the crushing displacement *x*.

**Peak crushing force (*PCF*):** This is the maximum value of load in the stable crushing stage.

**Mean crushing force (*P_mean_*):** This is the mean value of load in the stable crushing stage, which is expressed as:

(2) $$P_{mean}=\frac{EA}{d}$$

where *d* is the crushing distance.

**Specific energy absorption (*SEA*):** This is the energy absorbed per unit mass, which represents the energy-absorption efficiency and is expressed as:

(3) $$SEA=\frac{EA}{\mu d}=\frac{P_{mean}}{\mu }$$

where *μ* is the mass per unit length of the specimen.

**Crushing force efficiency (*CFE*)**: This is the ratio of mean crushing force to peak crushing force, which represents the consistency of load during crushing. Higher crushing force efficiency means a higher mean crushing force value and a lower peak crushing force. The *CFE* is expressed as:

(4) $$CFE=\frac{P_{mean}}{PCF}\times 100\%$$

## 3. Results and Discussion

### 3.1. Axial Crushing Response of Corresponding Individual Tubes

**Figure 5:**
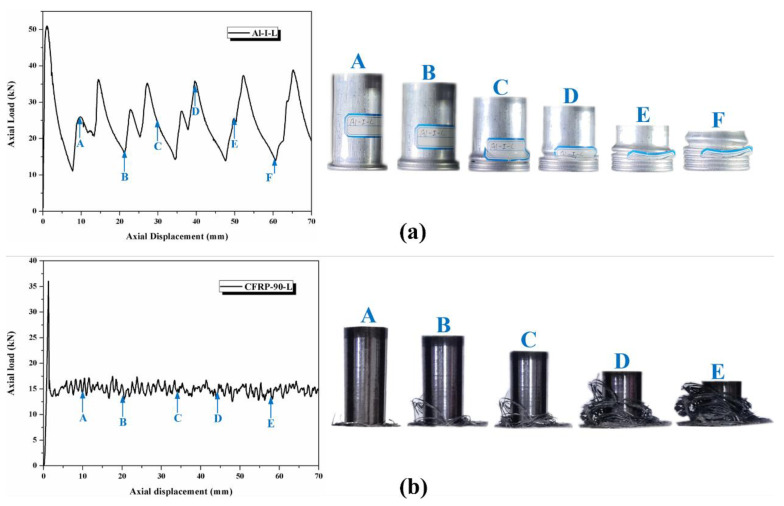
Load–displacement curves and crushing process of (**a**) aluminum alloy circular and (**b**) CFRP tubes.

To facilitate the comparison and subsequent analysis of the results, we first examine the energy-absorbing characteristics of corresponding individual tubes in the hybrid tubes. The crushing characteristics of the corresponding individual tubes are described using specimens Al-I-L and CFRP-90-L because the crushing characteristics of the same material in this test were the same. Further, the load–displacement curves of other specimens are discussed. Figure 5 shows that the axial crushing curves of the aluminum alloy and CFRP tubes can be divided into two stages: the stable loading stage before peak crushing force and the stable crushing stage after peak crushing force. Figure 5a illustrates the morphology of the aluminum alloy tube with a 10 mm displacement increase (as marked from point A to F). The load–displacement curve fluctuates frequently in the stable crushing stage, and each wave peak formed on the curve is accompanied by a symmetric concertina buckling fold. This deformation enables the aluminum alloy tube to absorb the axial crushing energy stably. The oscillation amplitude of the load–displacement curve of the CFRP tube is small in the stable crushing stage. Figure 5b shows that the specimen first underwent crushing failure at the bottom. The outer circumferential fiber layer continued to disintegrate as the displacement increased, causing many tiny fragments to fall to the bottom. A continuous cracking sound was heard during the experiment. The CFRP tubes mainly depended on the progressive crushing cracking of the fiber and matrix as well as the delamination between layers to dissipate the crushing energy. The progressive cracking mode was formed within a small range that caused the CFRP tubes to have a relatively stable crushing load level.

**Figure 6:**
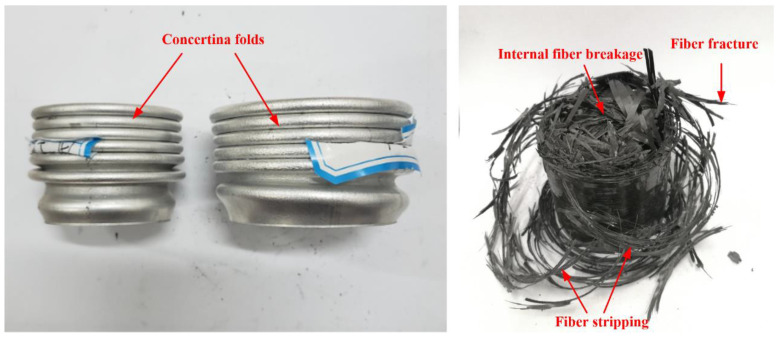
Crushing failure modes of aluminum alloy and CFRP tubes.

Figure 6 shows the typical failure mode of the aluminum alloy and CFRP tubes. Many symmetrical plastic buckling folds superimposed on each other were formed in the aluminum alloy tube, resulting in a “concertina” symmetrical deformation. The CFRP tube experienced many failure modes. Under the effects of axial compression and radial expansion of the circular tube, the outermost circumferential fiber layer experienced matrix cracking and fiber fracture. Owing to the restraint of the outer circumferential fibers, the inner fibers deformed and cracked radially inward, forming many small fragments inside the circular tube, and the fiber layer was fully broken.

### 3.2. Axial Crushing Response of 1/1 Hybrid Tube

**Figure 7:**
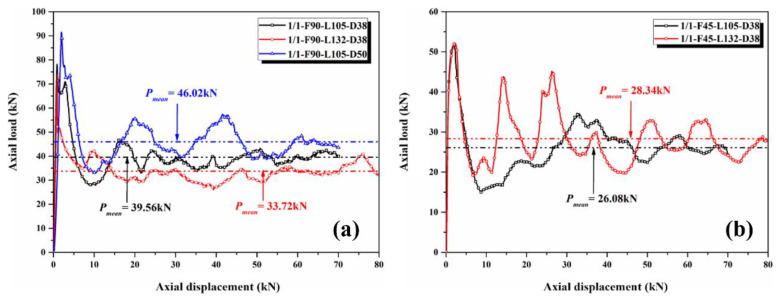
Load–displacement curves of 1/1 hybrid tubes with (**a**) [0°/90°] lay-up sequence and (**b**) [45°/−45°] lay-up sequence.

Generally, the axial crushing failure modes of the CFRP tubes can be divided into Mode I (progressive splaying mode), Mode II (progressive brittle fracturing mode), Mode III (progressive transverse shearing mode), Mode IV (progressive local buckling mode), Mode V (unstable local buckling mode), and Mode VI (medium length collapse mode) [22,28]. Figure 7 shows the load–displacement curves of the two kinds of 1/1 hybrid tubes with different fiber lay-up sequences. For the hybrid tubes with [0°/90°] fiber lay-up sequence, the *PCF* of specimens 1/1-F90-L105-D38 and 1/1-F90-L132-D38 was approximately 75 kN, and the trends of both curves in the crushing stage were similar, whereas that of the *P_mean_* was slightly different. Generally, the influence of length on the overall crushing response was not obvious. The *PCF* and *P_mean_* of specimen 1/1-F90-L120-D50 were considerably higher than those of the former two, and an increase in outer diameter had an obvious effect on the improvement of the crushing bearing performance. The *PCF* and *P_mean_* of the hybrid tubes with [45°/−45°] lay-up sequence were lower than those of the hybrid tubes with [0°/90°] lay-up sequence, and the fluctuation of the curves increased considerably in the stable crushing stage. The trends of the load–displacement curve of the two specimens with different lengths were different, indicating a certain change in the failure mode during the crushing process.

**Figure 8:**
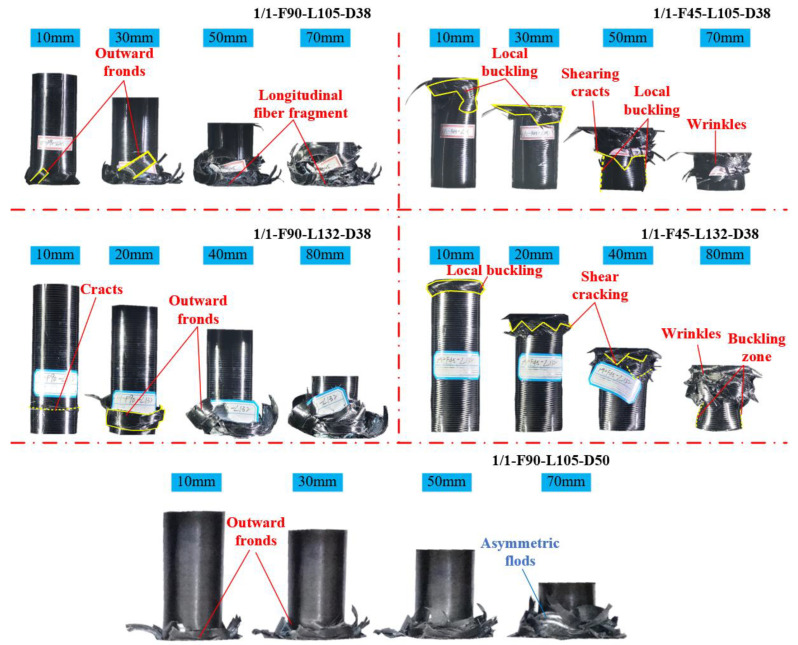
Axial crushing process of 1/1 hybrid tube.

Figure 8 illustrates the crushing morphology of each specimen with every 10–20 mm increase in displacement. The crushing processes of the three hybrid tubes with different sizes and [0°/90°] lay-up sequences were the same. The failure first occurred near the bottom, and the outer CFRP tube cracked under the influence of the buckling deformation of the inner aluminum alloy tube, forming long striping fragments. The failure region at the bottom of the CFRP tube continued to expand upward as the crushing displacement increased. The fiber fragments appeared to curl up because of the friction between the tube wall and the indenter. For specimens 1/1-F90-L105-D38 and 1/1-F90-L105-D50, the CFRP tube showed a progressive brittle fracturing mode (Mode II), the crushing failure mode of specimen 1/1-F90-L132-D38 changed slightly, and the cracking of the CFRP layer first occurred at approximately 26 mm from the bottom. The fragments of the CFRP tube were larger than those of specimen 1/1-F90-L105-D38; therefore, its *P_mean_* was slightly lower than that of specimen 1/1-F90-L105-D38.

The crushing processes of the two hybrid tubes with [45°/−45°] lay-up sequence differed. Figure 8 shows that when the crushing displacement of specimen 1/1-F45-L105-D38 reached approximately 10 mm, the external CFRP tube first generated an obvious local buckling at the top, which was approximately one-third of the total length. Then, the local buckling caused the matrix cracking of the CFRP tube. The matrix cracking continued to expand along 45° as the loading displacement increased, and the cracked matrix did not cause the fragments to fall off but they were gradually stacked on one another to form wrinkles. When the displacement increased to 60 mm, local buckling occurred again. Overall, the external CFRP tube of specimen 1/1-F45-L105-D38 showed an unstable local buckling mode (Mode V) during the crushing process. Specimen 1/1-F45-L132-D38 first had a local buckling failure at the top, but the buckling range with an approximately symmetrical circular shape was small. Before the crushing displacement increased to 40 mm, the external CFRP tube showed a slowly downward expanding matrix shear cracking and gradually formed wrinkles. The remaining main tube wall did not change considerably, and the crushing failure mode was relatively stable. Therefore, the crushing load of specimen 1/1-F45-L132-D38 before the displacement reached 40 mm was slightly higher than that of specimen 1/1-F45-L105-D38. When the displacement increased to approximately 50 mm, the tube wall showed an unstable local buckling, and the trend of the load–displacement curve of specimen 1/1-F45-L132-D38 was consistent with that of specimen 1/1-F45-L105-D38. The main crushing failure mode of the CFRP tube in specimen 1/1-F45-L132-D38 was a mixed mode (Mode IV and Mode V), which was first demonstrated as stable buckling deformation folding and then unstable buckling deformation.

**Figure 9:**
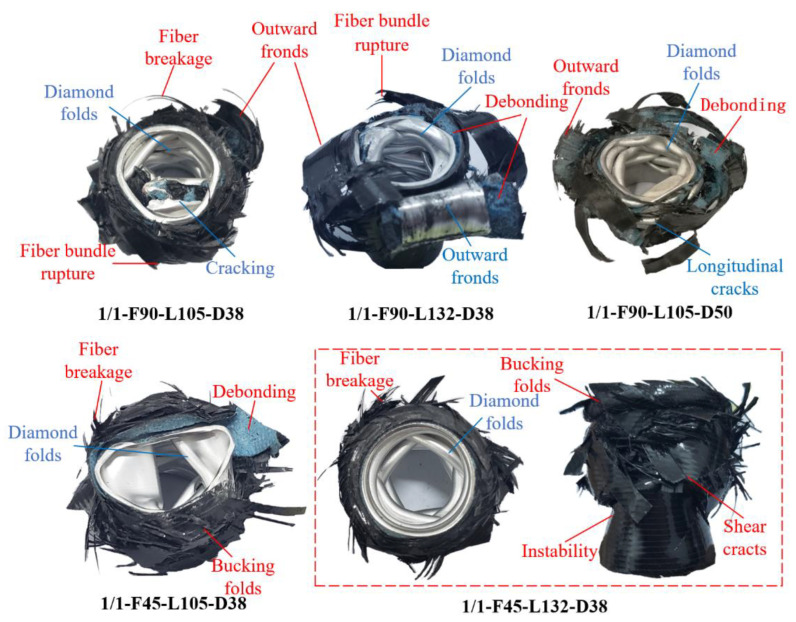
Final failure modes of 1/1 hybrid tubes with two lay-up sequences.

The final failure modes of the specimens were presented in Figure 9. Under the outer CFRP tube restraint, the inner aluminum alloy tubes of all the specimens were in the asymmetric diamond buckling mode unlike the concertina bucking mode of the single aluminum alloy tube. The inner aluminum alloy tube of specimens 1/1-F90-L105-D38 and 1/1-F90-L132-D38 also experienced obvious plastic cracking. The failure mode of the outer CFRP tube of the hybrid tube varied with the size and lay-up sequence of the specimens. For specimens with the [0°/90°] lay-up sequence, the main failure mode of the CFRP layer was fiber bundle fracturing and matrix transverse cracking, forming many whole pieces of fragments in the tubes. For specimens with the [45°/−45°] lay-up sequence, the CFRP tubes showed a buckling and folding deformation mode, accompanied by a high amount of matrix shear cracking, and only a few fibers broke. The fibers contributed minimally to bearing the crushing load. Additionally, the debonding failure between the aluminum alloy and CFRP tubes was found in all the specimens. Overall, the energy dissipation modes of the hybrid tubes with the [0°/90°] lay-up sequence mainly included plastic deformation of aluminum alloy, brittle fracturing and curling of the CFRP layer, debonding between layers, and friction among components, specimens, and the indenter. The energy dissipation of the hybrid tubes with the [45°/−45°] lay-up sequence mainly depended on the matrix shear failure, the plastic folding of aluminum alloy, debonding between layers, and friction between components and between the specimen and the indenter.

### 3.3. Axial Crushing Response of 2/1 Hybrid Tube

**Figure 10:**
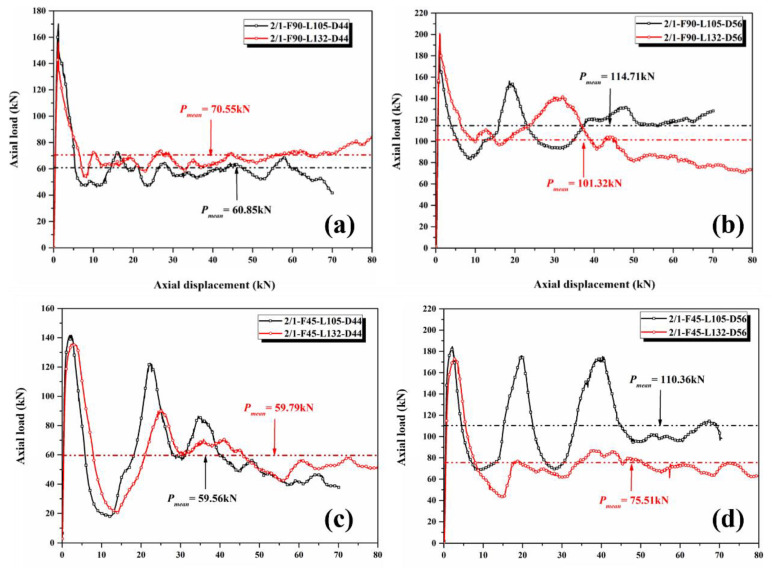
Load–displacement curve of 2/1 hybrid tubes: [0°/90°] lay-up sequence with a nominal outer diameter of (**a**) 44 mm and (**b**) 56 mm; [45°/−45°] lay-up sequence with a nominal outer diameter of (**c**) 44 mm and (**d**) 56 mm.

Figure 10 shows the comparison chart of the load–displacement curves of the 2/1 hybrid tubes. Similar to the 1/1 hybrid tube, the *PCF* and *P_mean_* of specimens with [0°/90°] lay-up sequence were higher than those of specimens with [45°/−45°] lay-up sequence under the same geometric size. The effects of specimen length on the load–displacement response of specimens with different outer diameters varied. Figure 10a,c show that changes in the specimen length had a relatively minimal effect on the curve trend in the stable crushing stage while the outer diameter of the specimen was 44 mm. The oscillation amplitude of the load–displacement curve of the hybrid tube with the [0°/90°] lay-up sequence was small during the crushing process, and the overall load level was relatively stable. The curve of the hybrid tube with the [45°/−45°] lay-up sequence showed two obvious fluctuations before the crushing displacement reached 50 mm, and then the load level remained stable. However, the *P_mean_* of specimen 2/1-F90-L132-D44 was 16% more than that of specimen 2/1-F90-L105-D44, and the *P_mean_* of specimen 2/1-F45-L132-D44 was the same as that of specimen 2/1-F45-L105-D44. The influence of specimen length on the load–displacement curve of the specimens with a nominal outer diameter of 56 mm was relatively significant. Figure 10b,d show that in the stable crushing stage, the curves of specimens 2/1-F90-L132-D56 and 2/1-F90-L105-D56 showed large fluctuations, and the *P_mean_* of the former was approximately 11.7% lower than that of the latter. The curve trends of specimens 2/1-F45-L132-D56 and 2/1-F45-L105-D56 were particularly different. The oscillation amplitude of the load–displacement curves of the former in the crushing stage was small, and the *P_mean_* was 75.51 kN. The curve of the latter in the crushing stage fluctuated drastically, and the *P_mean_* reached 110.36 kN. This phenomenon was because the length–diameter ratio of most specimens was between 2 and 3, whereas the length–diameter ratio of the hybrid tube with a nominal outer diameter of 56 mm and length of 105 mm was only 1.84. The stress concentration at the loading surface was severe, causing a change in the crushing mode.

**Figure 11:**
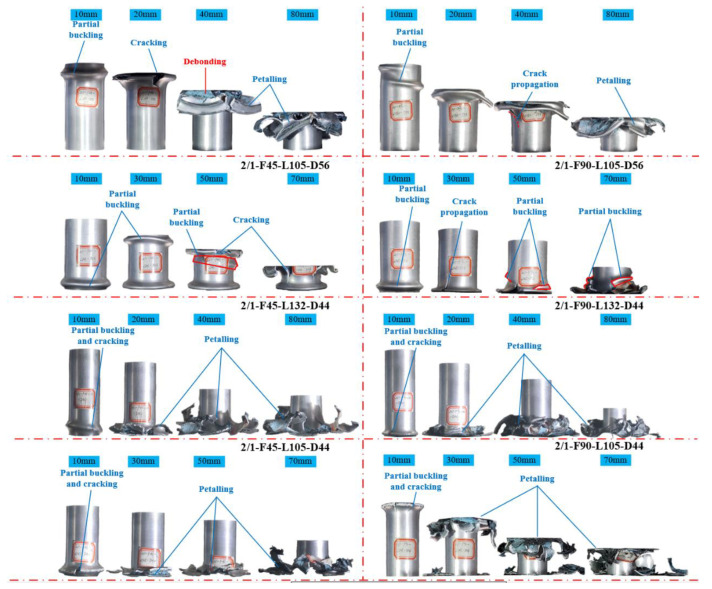
Axial crushing deformation process of 2/1 hybrid tube.

Figure 11 illustrates the differences between the crushing processes of each specimen. The outer aluminum alloy tube of the 2/1 hybrid tube did not undergo progressive folding deformation because it was hindered by the inner CFRP tube. However, the aluminum alloy tube showed a failure mode similar to splitting and curling. The crushing failure processes of each specimen were approximately the same: the outer aluminum alloy tubes first experienced “elephant-foot buckling” near the top or bottom of the tubes. As crushing displacement increased, the buckling zone of the outer aluminum alloy tube cracked, the middle CFRP tube underwent progressive crushing failure, and the inner aluminum alloy tube underwent “diamond” plastic folding. Under the action of the friction between the specimen and indenter, the outer aluminum alloy tube continuously turned outward and gradually broke into pieces. Comparing the two specimens with different outer diameters, the failure of the specimens with a nominal outer diameter of 44 mm started from the end and gradually expanded along the axial direction of the specimens. The crushing process was relatively stable; consequently, the change characteristics of the curves of each specimen in the stable crushing stage were similar. The crushing failure process of the specimen with a nominal outer diameter of 56 mm varied with length. The crushing failure characteristics of specimens 2/1-F45-L132-D56 and 2/1-F90-L132-D56 were similar to those of specimens with a nominal outer diameter of 44 mm. During the entire process, the outer aluminum alloy tube only experienced a buckling fold after the peak load and then cracked. The outer aluminum alloy tube of specimen 2/1-F45-L105-D56 first underwent a plastic buckling fold at the bottom after the peak load point. When the displacement reached approximately 20 mm and 40 mm, the second and third plastic buckling folds appeared successively at the top end, and then cracks occurred, corresponding to two obvious oscillations in the load–displacement curve. A similar phenomenon also occurred in specimen 2/1-F90-L105-D56 when the displacement was approximately 20 mm and 50 mm. However, when the specimen was affected by different lay-up sequences of the internal CFRP layer, longitudinal cracking occurred and expanded rapidly after buckling folds were formed. Therefore, the peak value of the specimen at the two corresponding displacements was not uniform. These results show that when the length–diameter ratio was too small, the effect of stress concentration at the end of the test changed the deformation mode of the 2/1 hybrid tube, and the oscillation of the load–displacement curve was obvious in the crushing process. Therefore, it was difficult for the specimen to have a stable and controllable progressive crushing failure mode.

**Figure 12:**
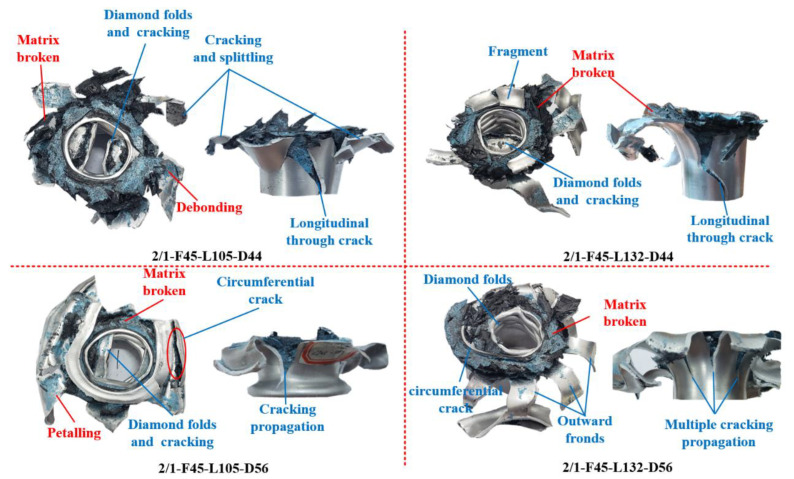
Final failure modes of 2/1 hybrid tubes with [45°/−45°] lay-up sequence.

Figure 12 shows the final failure mode of the specimens with [45°/−45°] lay-up sequence. The main crushing failure modes of the specimens included diamond buckling/fracture of the inner aluminum alloy tube, fracture and cracking of the middle CFRP tube, splitting and curling of the outer aluminum alloy tube, and delamination and debonding between layers. When the nominal outer diameter was 44 mm, the failure mode of the specimens with the [45°/−45°] lay-up sequence was not affected by length. A longitudinal crack was found on the outer aluminum alloy tube of specimens 2/1-F45-L132-D44 and 2/1-F45-L105-D44; therefore, the crushing load levels of the two specimens were similar. When the nominal outer diameter increased to 56 mm, the longitudinal non-through and circumferential cracks along the initial position of the “elephant-foot buckling” folds appeared on the outer aluminum alloy tube of the specimen with the [45°/−45°] lay-up sequence. The longitudinal cracks of the outer aluminum alloy tube of specimen 2/1-F45-L132-D56 were more evenly distributed, the cracks were more, and the crack propagation path was straighter, causing the outer aluminum alloy tube to experience a splitting and curling failure mode. Figure 10d shows that compared with buckling and folding failure, the splitting and curling failure of the aluminum alloy tube caused the fluctuation of the load–displacement curve to be smaller, increased the crushing displacement, and caused the circumferential crack to divide the specimen into upper and lower parts. This phenomenon destroyed the integrity of the outer aluminum alloy tube and sustained the deformation, causing an unsatisfactory cracking mode. It is necessary to resolve the circumferential cracking problem using a reasonable trigger mechanism at the end. The failure mode of the CFRP tube with the [45°/−45°] lay-up sequence was mainly matrix shear cracking, and the failure mode of the CFRP layer of each specimen was consistent.

**Figure 13:**
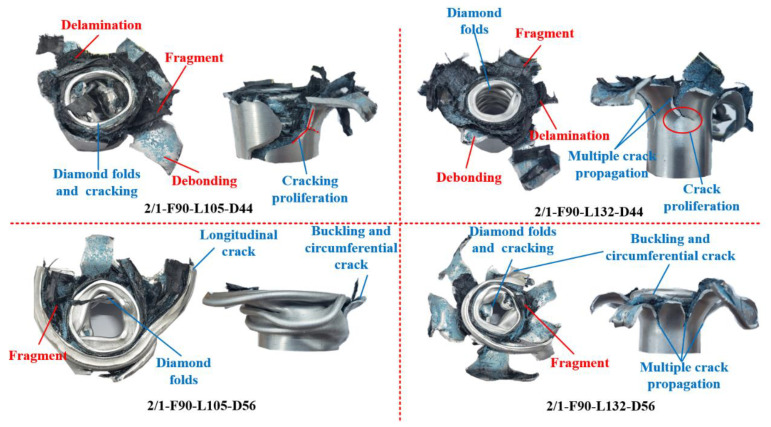
Final failure modes of 2/1 hybrid tubes with [0°/90°] lay-up sequence.

When the nominal outer diameter was 56 mm, the failure mode of the outer aluminum alloy tube of the specimens with the [0°/90°] lay-up sequence was the same as that of the specimens with the [45°/−45°] lay-up sequence, as presented in Figure 13. For specimens with a nominal outer diameter of 44 mm and the [0°/90°] lay-up sequence, the outer aluminum alloy tube of specimen 2/1-F90-L105-D44 experienced longitudinal through cracking; hence, the load–displacement curve continued to decline in the later stage. The crack of the outer aluminum alloy tube of specimen 2/1-F90-L132-D44 caused the proliferation of single cracks and the formation of multiple cracks during the cracking propagation process, and approximate tearing and curling were also obvious. The load–displacement curve had an upward trend in the later stage of crushing; therefore, the *P_mean_* was higher than that of specimen 2/1-F90-L105-D44. The failure modes of the CFRP tubes differed because of the different failure modes of the aluminum alloy outer tubes. The CFRP tube of specimen 2/1-F90-L105-D44 showed progressive shear crushing failure, whereas the CFRP tube of specimen 2/1-F90-L132-D44 formed progressive outward turning crushing failure. Overall, the energy absorption of the 2/1 hybrid tube mainly depended on the plastic deformation and cracking of the outer aluminum alloy tube, the crushing failure of CFRP in the middle layer, the plastic deformation of the inner aluminum alloy tube, and the delamination and debonding between the layers and friction.

### 3.4. Comparison of Typical Energy-Absorption Indicators

**Figure 14:**
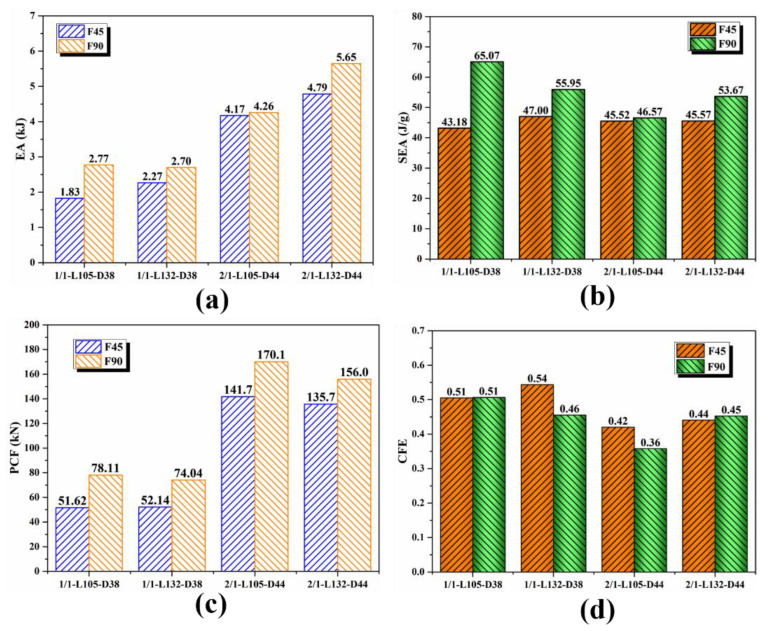
Comparison of energy-absorption performance between 1/1 and 2/1 hybrid tubes: (**a**) *EA*; (**b**) *SEA*; (**c**) *PCF*; (**d**) *CFE*.

The energy-absorption effect of the 1/1 and 2/1 hybrid tubes was compared as shown in Figure 14a,b. It can be seen from Table 1 that the 2/1 hybrid tube was composed of the 1/1 hybrid tube and a layer of aluminum alloy tube on the outside. For the two types of hybrid tubes, the *EA* and *SEA* of the specimens with the [0°/90°] lay-up sequence were higher than that of the specimens with the [45°/−45°] lay-up sequence, indicating better energy-absorption performance. The total energy absorption of the 2/1 hybrid tubes was higher than that of the 1/1 hybrid tubes owing to an increase in cross-sectional area, but the relationship between the *SEA* of the two specimens varied with the lay-up sequence and length. When the [45°/−45°] lay-up sequence was applied, the *SEA* of the 1/1 and 2/1 hybrid tubes was approximately the same. This phenomenon occurred because the main failure mode of the fiber layer was the shear failure and matrix folding, and the fiber did not fully serve its function. When the [0°/90°] lay-up sequence was applied, the *SEA* of the 1/1 hybrid tubes with a length of 105 mm was approximately 39.7% higher than that of the 2/1 hybrid tubes. The overall cracking of the outer aluminum alloy tube in the 2/1 hybrid tube reduced the specimen’s energy-absorption effect. When the specimen length increased to 132 mm, the difference was reduced to 4.2%, and the *SEA* of the two specimens was the same. Figure 14c,d presents the comparison of *PCF* and *CFE* of the specimens. Although the *P_mean_* of the specimen with the [0°/90°] lay-up sequence was higher than that of the specimen with the [45°/−45°] lay-up sequence, the former did not show any advantage over the latter in terms of *CFE* because of its high *PCF*.

**Figure 15:**
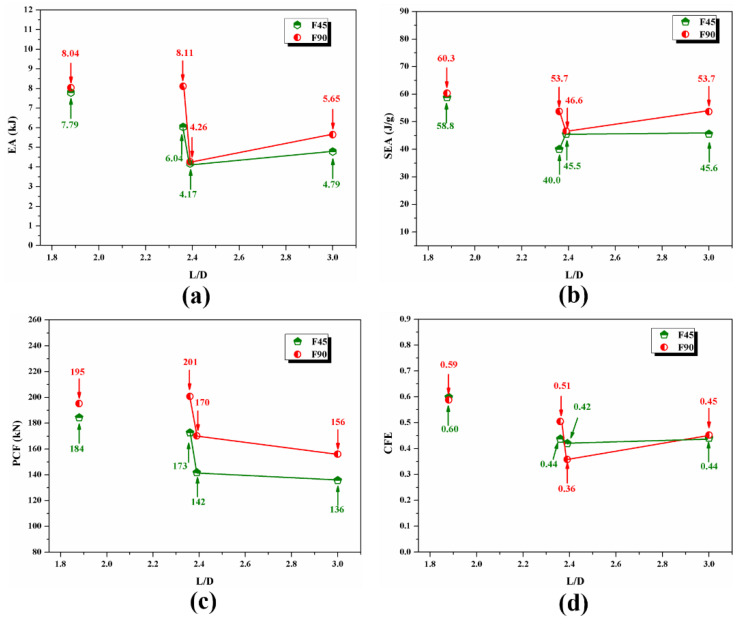
Effects of geometric size on the energy-absorption performance of 2/1 hybrid tube: (**a**) *EA*; (**b**) *SEA*; (**c**) *PCF*; (**d**) *CFE*.

The energy-absorption effects of the 2/1 hybrid tubes with different geometric sizes were further compared in Figure 15. The change in the specimens’ geometric size was characterized by the length–diameter ratio *L*/*D*. The two specimens with a length–diameter ratio of 1.84 were not listed as comparative objects because they did not undergo stable crushing failure. Comparing the crushing energy-absorption performance indexes of each specimen, we found that under a consistent and stable crushing failure mode, the larger the outer diameter of the specimen, the longer the crushing displacement, and the higher the energy absorption. *SEA* differed slightly because the thickness ratios of the aluminum alloy and fiber layers of the two specimens with different outer diameters were the same, indicating that *SEA* was less affected by geometric size. Comparing the indexes of crushing performance, we found that the *PCF* was less affected by length, and the specimen with a large outer diameter had higher a *PCF*. Additionally, the *CFE*s of specimens with different diameters and lengths were similar. These results show that the relative indexes of the energy-absorption performance (*SEA*, *CFE*) of the 2/1 hybrid tube was not considerably affected by geometric size under stable crushing, indicating that the quality of the hybrid tubes formed by the spinning process was relatively stable.

**Figure 16:**
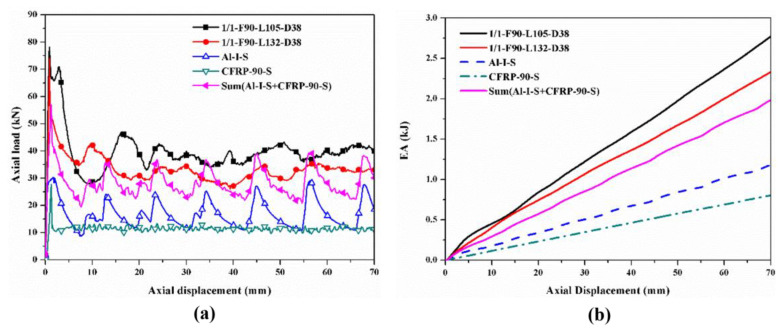
Hybrid effect of 1/1 hybrid tube with nominal outer diameter of 38 mm: (**a**) axial load–displacement curves; (**b**) *EA*–displacement curves.

**Figure 17:**
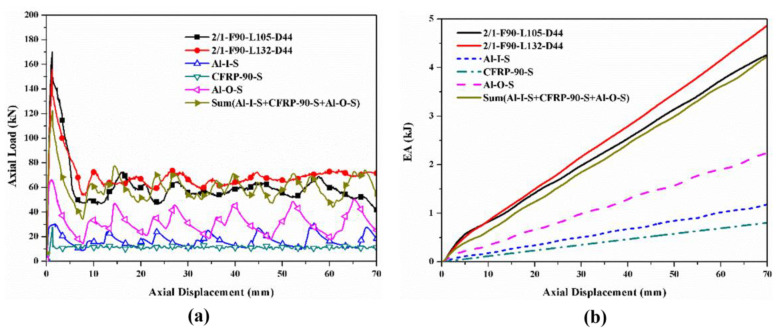
Hybrid effect of 2/1 hybrid tube with nominal outer diameter of 44 mm: (**a**) axial load–displacement curves; (**b**) *EA*–displacement curves.

**Figure 18:**
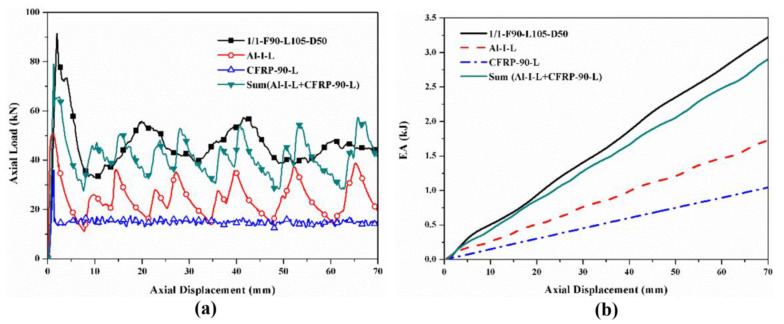
Hybrid effect of 1/1 hybrid tube with nominal outer diameter of 50 mm: (**a**) axial load–displacement curves; (**b**) *EA*–displacement curves.

**Figure 19:**
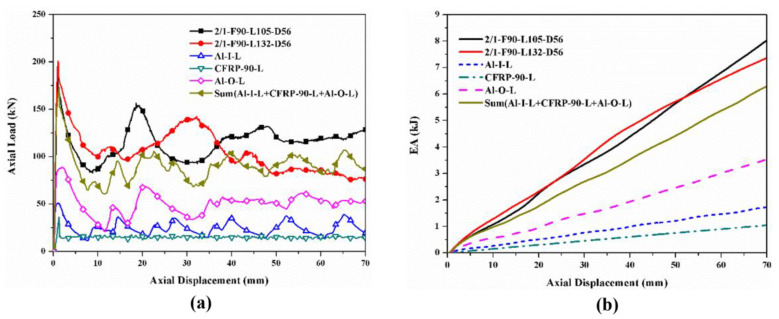
Hybrid effect of 2/1 hybrid tube with nominal outer diameter of 56 mm: (**a**) axial load–displacement curves; (**b**) *EA*–displacement curves.

The hybrid effects were investigated by comparing the sum of the load–displacement curves and *EA*–displacement curves of the corresponding individual tubes with those of the hybrid tubes, which are shown in Figure 16, Figure 17, Figure 18 and Figure 19. Only the specimens with the [0°/90°] lay-up sequence were considered here as a case study since they demonstrated a better comprehensive energy-absorption effect. The crushing distance was selected as 70 mm. The energy-absorption curves of the two types of hybrid tubes with different stacking configurations were higher than the sum of the energy-absorption curves of all the corresponding individual tubes. As the displacement increased, the difference between the energy-absorption of the hybrid tubes and the sum of the energy-absorption of all the corresponding individual tubes gradually decreased, showing that the energy-absorption performance of the corresponding individual tubes had an effect of “1 + 1 > 2” after combining with the tubes through the adhesive layer to form a hybrid tube. Considering the configurational characteristics and deformation mode of the hybrid tube, we found in the 1/1 hybrid tubes that adding the external CFRP tube improved the bending stiffness of the inner aluminum alloy tube, enhanced the ability of the aluminum alloy tube to resist local buckling, and thus improved the overall energy-absorption effect of the specimen [27]. In the 2/1 hybrid tubes, the middle CFRP pipe had an enhancing effect on the inner aluminum alloy tube, which was similar to that of the 1/1 hybrid tubes, and the outer aluminum alloy tube was affected by the deformation of the middle CFRP tube, failure mode from the local plastic buckling, and folding failure to tearing and curling failure. The deformation of the inner and outer aluminum alloy tubes also changed the failure mode of the middle CFRP tube. Therefore, under the interaction between layers, the energy absorption of the aluminum/CFRP hybrid tubes had a positive hybrid effect.

Further, according to Sun et al. [12], the hybrid effect ratio *S_e_* was introduced to evaluate the hybrid effect of the energy absorption of hybrid tubes. The hybrid effect ratio *S_e_* is expressed as follows:

(5) $$\begin{array}{l} EA_{int}=EA_{hybrid}-(EA_{Al}+EA_{CFRP})\\ S_{e}=\frac{EA_{int}}{EA_{Al}+EA_{CFRP}}\times 100\% \end{array}$$

where *EA_hybrid_* is the energy-absorption of the hybrid tube; *EA_Al_* and *EA_CFRP_* are the energy-absorption of the single aluminum alloy and CFRP tubes, respectively; and *EA_int_* is the surplus energy-absorption of the hybrid tube under the influence of the hybrid effect.

**Figure 20:**
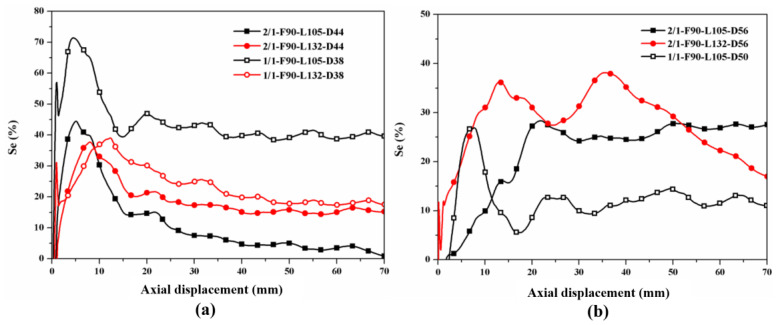
Comparison of hybrid effect ratio between the tubes: (**a**) specimens with large *L*/*D* ratio; (**b**) specimens with small *L*/*D* ratio.

Figure 20 shows the curves of the hybrid effect ratio of the 1/1 and 2/1 hybrid tubes. For the specimens with a smaller outer diameter, the hybrid effect of the 1/1 hybrid tube with a length of 105 mm was the most prominent, and the hybrid effect ratio remained at approximately 40% in the stable crushing stage. For the 2/1 hybrid tube with a length of 105 mm, the hybrid effect ratio was almost reduced to zero in the later crushing stage because of the overall cracking of the aluminum alloy outer tube. When the length increased to 132 mm, the hybrid effect ratio decreased because the outside CFRP layer of the 1/1 hybrid tubes experienced large cracking fragments. The failure mode of the 2/1 hybrid tubes was relatively stable, and the hybrid effect ratio was the same as that of the 1/1 hybrid tubes, and the ratio was approximately 15–20%. For the specimens with a larger outer diameter, the hybrid effect ratio of specimen 2/1-F90-L105-D56 was stable at approximately 25%, which was higher than that of specimen 1/1-F90-L105-D50 by 10%. The hybrid effect ratio of specimen 2/1F90-L132-D56 changed considerably, decreasing substantially after a displacement of 40 mm and remaining lower than that of specimen 2/1-F90-L105-D56 after 50 mm; these changes correspond to the decrease in *EA* in Figure 19.

## 4. Conclusions

This study systematically explored the effects of geometry size and lay-up sequence on the energy-absorption performance and crushing failure mode of 2/1 and 1/1 hybrid tubes through axial crushing tests. The experimental results showed that in the 1/1 and 2/1 hybrid tubes, the energy-absorption effect of the specimen with the [0°/90°] lay-up sequence was better than that of the specimens with the [45°/−45°] lay-up sequence. When the lengths of the specimens were too low and affected by the stress concentration at the end, it was difficult for the specimens to maintain a stable and controllable progressive crushing failure mode, which increased the randomness of the crushing failure modes, and the fluctuation of the load–displacement curves. When the crushing failure process was relatively stable, the specific energy absorption and crushing force efficiency of the 2/1 hybrid tubes were not affected by the geometric size. The stable crushing mode of the 1/1 and 2/1 hybrid tubes was that the aluminum alloy tube first experienced buckling near the end, and then each layer continued to crack from the buckling position. The failure mode of the 1/1 hybrid tube was mainly diamond plastic folding, inner aluminum alloy tube cracking, and outer CFRP tube cracking. The failure mode of the 2/1 hybrid tube was mainly plastic folding, inner aluminum alloy tube cracking, progressive crushing failure of the CFRP layer, and tearing and curling of the outer aluminum alloy tube. The adhesive layer changed the failure modes of the corresponding individual tubes of the 1/1 and 2/1 hybrid tubes. Therefore, the energy absorption of the hybrid tubes was higher than the sum of the energy absorption of all the corresponding individual tubes, showing a positive hybrid effect and the hybrid effect of the hybrid tube was investigated by comparing the sum of the energy absorption of all corresponding individual tubes and the energy absorption of the hybrid tube.

## Figures and Tables

**Table 1:** Geometrical parameters of specimens in axial crushing test.

Sample Code	Stacking Structure	Stacking Configuration	Length *L* (mm)	Outer Diameter *D* (mm)	Thickness of Al *t_m_* (mm)	Thickness of CFRP *t*_c_ (mm)	Mass *m* (g)	*L*/*D*
2/1-F90-L132-D44	2/1	[0°/90°]_3_	132	44.62	3.11	0.90	173.7	2.96
2/1-F45-L132-D44	2/1	[45°/−45°]_3_	132	44.62	3.11	0.90	173.3	2.96
2/1-F90-L105-D44	2/1	[0°/90°]_3_	105	44.62	3.11	0.90	137.2	2.35
2/1-F45-L105-D44	2/1	[45°/−45°]_3_	105	44.62	3.11	0.90	137.5	2.35
2/1-F90-L132-D56	2/1	[0°/90°]_3_	132	57.22	3.14	0.92	249.0	2.31
2/1-F45-L132-D56	2/1	[45°/−45°]_3_	132	57.22	3.14	0.92	249.0	2.31
2/1-F90-L105-D56	2/1	[0°/90°]_3_	105	57.22	3.14	0.92	199.8	1.84
2/1-F45-L105-D56	2/1	[45°/−45°]_3_	105	57.22	3.14	0.92	198.7	1.84
1/1-F90-L132-D38	1/1	[0°/90°]_3_	132	40.45	1.38	0.99	79.6	3.26
1/1-F45-L132-D38	1/1	[45°/−45°]_3_	132	40.45	1.38	0.99	79.6	3.26
1/1-F90-L105-D38	1/1	[0°/90°]_3_	105	40.45	1.38	0.99	63.9	2.60
1/1-F45-L105-D38	1/1	[45°/−45°]_3_	105	40.45	1.38	0.99	63.5	2.60
1/1-F90-L105-D50	1/1	[0°/90°]_3_	105	52.38	1.55	0.99	93.9	2.00
Al-I-S	—	—	105	38.07	1.38	—	43.5	—
Al-I-L	—	—	105	49.99	1.55	—	65.8	—
Al-O-S	—	—	105	45.09	2.07	—	76.2	—
Al-O-L	—	—	105	57.21	2.30	—	105.9	—
CFRP-90-S	—	[0°/90°]_3_	105	40.3	—	1.11	17.5	—
CFRP-90-L	—	[0°/90°]_3_	105	52.21	—	1.11	28.1	—

## Data Availability

Data available on request due to restrictions eg privacy or ethical.
